# Comparison of Robot-assisted Enhanced-view Totally Extraperitoneal (eTEP) and Transabdominal Retromuscular (TARM aka TARUP) Ventral Hernia Mesh Repair: A Systematic Review and Meta-Analysis

**DOI:** 10.3389/jaws.2025.14723

**Published:** 2025-07-04

**Authors:** Francesco Brucchi, Annabelle De Troyer, Richard Sassun, Gianlorenzo Dionigi, Filip Muysoms

**Affiliations:** 1 General Surgery Residency Program, University of Milan, Milan, Italy; 2 Division of Surgery, Istituto Auxologico Italiano, Istituto di Ricovero e Cura a Carattere Scientifico (IRCCS), Milan, Italy; 3 Department of General Surgery, Ziekenhuis Aan de Stroom (ZAS) Antwerp, Antwerp, Belgium; 4 Colorectal Surgery Unit, Fondazione IRCCS Istituto Nazionale Dei Tumori, Milan, Italy; 5 Department of Pathophysiology and Transplantation, University of Milan, Milan, Italy; 6 Department of Surgery, Maria Middelares Ghent, Ghent, Belgium

**Keywords:** robot-assisted, robotic, eTEP, TARM, TARUP

## Abstract

**Background:**

This systematic review and meta-analysis compares robotic eTEP and TARM/TARUP in terms of complications, operative time, infections, length of stay, seroma, and short-term recurrence rates.

**Methods:**

A systematic review was conducted following PRISMA guidelines, searching MEDLINE, Embase, and CENTRAL until January 30, 2025. Studies comparing r-eTEP and r-TARM/TARUP in adults with ventral hernia were included. Primary outcomes were operative time and postoperative complications. Secondary outcomes included wound complications, length of stay, readmission, pain, and short-term recurrence. A random-effects model was used for meta-analysis, and study quality was assessed via the Methodological Index for Non-randomised Studies (MINORS) score.

**Results:**

Three studies (308 patients: r-eTEP 176, r-TARM/TARUP 132) were included. Overall complications were lower with r-eTEP (RD: -0.17; 95% CI: -0.27 to −0.07; p = 0.001) and as was the case for minor complications (RD: -0.14; 95% CI: -0.22 to −0.06; p = 0.0008). No significant differences were found in major complications, SSI, recurrence, or 30-day readmission. Operative time was shorter with r-eTEP (MD: -25.66 min; 95% CI: -51.18 to −0.14; p = 0.05, I^2^ = 88%). Seroma formation was lower with r-eTEP (RD: -0.08; 95% CI: -0.15 to −0.02; p = 0.01). Length of stay was shorter with r-eTEP (MD: -2.64 days; 95% CI: -4.06 to −1.22; p = 0.004, I^2^ = 98%).

**Conclusion:**

Evidence remains insufficient to favor one robotic approach over the other. High-quality prospective studies on patient outcomes and long-term recurrence are needed to guide surgical decision-making.

**Systematic Review Registration:**

PROSPERO, identifier CRD420250650879.

## Introduction

The Rives-Stoppa technique has long been the gold standard for open repair of ventral midline hernias and offers a reliable approach to abdominal wall reconstruction [1]. A major advantage of this technique is the retromuscular mesh insertion, which has been associated with a lower recurrence rate, less surgical site infection, and improved abdominal wall function [2, 3].

Over time, laparoscopic adaptation of retromuscular approach evolved [4, 5] and paved the way for the integration of robotic-assisted technology [6], which offers improved precision, visualization and dexterity. Recent data from the Danish Hernia Database supports the use of robotic surgery in ventral hernia repair and demonstrates advantages over both open surgery and laparoscopic intraperitoneal mesh repair [7–9]. The robotic platform allows two primary access methods: the transabdominal (TA) approach, which includes a peritoneal entry, and the totally extraperitoneal (TEP) approach, which preserves the integrity of the peritoneum during access to the retromuscular space [10–12]. For these approaches a lateral docking [13–16] and caudal docking [17, 18] has been described.

While the TA approach is the conventional method for robotic retromuscular hernia repair, recent advances have led to the development of the extended (or enhanced) view totally extraperitoneal (eTEP) technique for robotic ventral hernia repair (VHR) [12, 19]. Despite the increasing acceptance of these techniques, comparative studies remain limited. Although there are numerous studies in the literature evaluating laparoscopic TAPP and extraperitoneal TEP approaches in minimally invasive inguinal hernia repair (IHR) [20], similar comparisons in ventral hernia repair (VHR), particularly using the same trocar approach, are scarce.

Both the robotic eTEP and transabdominal retromuscular (TARM) [21] techniques allow access to the retrorectus space, enabling a minimally invasive alternative of the Rives-Stoppa repair. However, each method offers unique advantages and potential risks, emphasizing the need for direct comparison of their outcomes.

This systematic review and meta-analysis aims to compare the robotic eTEP and TARM/TARUP methods, specifically evaluating overall, major, and minor complications, operative time, surgical site infection rates, seroma formation and short-term recurrence rates.

## Materials and Methods

### Data Sources and Research

The peer-reviewed literature published from January 1, 2000 to January 30, 2025 was searched in the Medline (PubMed), Embase, Scopus, and Cochrane Library databases. The following keywords were used to identify relevant studies: “eTEP,” “robotic,” “sublay,” “retromuscular,” “retrorectus,” “TARM,” “TARUP,” “mesh repair,” “robotic,” “hybrid,” “umbilical hernia,” “ventral hernia,” “epigastric hernia,” “incisional hernia.” The detailed search strategies can be found in the Supplementary Material (Supplementary Figure S1). This meta-analysis was conducted in accordance with the PRISMA (Preferred Reporting Items for a Systematic Review and Meta-analysis) [22] and AMSTAR II Statements (Supplementary Files S1, S2a, S2b). The planned protocol of this meta-analysis was registered in PROSPERO (PROSPERO 2025: CRD420250650879). In addition, reference lists of retrieved articles were screened to identify further studies.

### Selection of the Studies

Two reviewers (FB, RS) independently performed the literature search and data extraction using the Rayyan software for systematic review [23]. They independently assessed the eligibility of all preliminarily identified records first by the title and then by abstract. After screening, the full-text manuscripts of relevant studies were carefully read to confirm eligibility and extract useful information. Disagreements regarding the eligibility of articles were resolved by a third reviewer (GD). Studies were included according to the following criteria: 1) randomized and observational studies in English, 2) adult population diagnosed with ventral hernia, 3) repair using robotic extended view totally extraperitoneal (eTEP) versus transabdominal retromuscular (TARM) ventral hernia repair, 4) detailed description of surgical technique and report of short term results. No geographical restrictions were made. Reviews, editorials, case reports of <5 patients and manuscripts on other minimally invasive techniques were excluded. Patients undergoing concurrent procedures were excluded. Papers reporting duplicate results from the same author group were excluded.

### Data Extraction and Quality Assessment

Two authors examined the main characteristics of each article, found and provided the following data: Year of publication, country where the study was conducted and source of population, total number of subjects, sex, age, outcomes, length of primary hospitalisation (LOS), recurrence, duration of follow-up, effectiveness of treatment performed, statistical analysis. The methodological quality of the selected studies was assessed using the criteria of the Methodological Index for Non-randomised Studies (MINORS) [24]. The assessment of the bias of the included studies is listed in the Supplementary Material (Supplementary Table S1). The overall quality of the evidence was assessed using the GRADE (Grading of Recommendations, Assessment, Development and Evaluations) approach [25]. Based on the overall assessment the quality of the evidence was categorised into four levels (high, moderate, low or very low). Studies were either downgraded or upgraded in quality depending on whether the criteria of risk of bias, inconsistency, indirectness, imprecision, publication bias, large magnitude, dose dependence or effect of all plausible confounders were met. Authors FB and RS performed the GRADE assessment.

### Outcome Measures

Primary outcomes, including:

- Postoperative total, minor and major complications and operative time

Complications were classified as minor or major based on the Clavien-Dindo classification system. Grades I and II were categorized as minor complications, while Grades III and IV were classified as major complications.

Secondary outcomes, including:

- Wound complications (surgical site infection, seroma), length of stay (LOS), short-term recurrence rate, 30-day readmission, postoperative pain.

### Data Synthesis and Analysis

Risk Difference (RD) was selected as the effect measure for binary outcomes because of its clinical interpretability and its appropriateness in studies with small sample sizes and low event rates. It was calculated for discrete variables with 95% confidence intervals (c.i.) calculated using a Mantel–Haenszel random-effects model. Mean Difference (MD) were calculated for continuous variables with 95% c.i. using an inverse-variance random-effects model. The random-effects model was applied to all analyses, regardless of I^2^ values, in order to account for potential clinical and methodological heterogeneity among studies.

Heterogeneity was assessed using Cochran’s Q test and the I^2^ statistic: I^2^ <25% = Low heterogeneity, I^2^ 25%–50% = Moderate heterogeneity, I^2^ >50% = High heterogeneity. All statistical analyses were performed using Revman software, version 5.4.1 (Cochrane Collaboration, The Nordic Cochrane Centre, Copenhagen), with significance set at p < 0.05.

## Results

### Search Results

**FIGURE 1:**
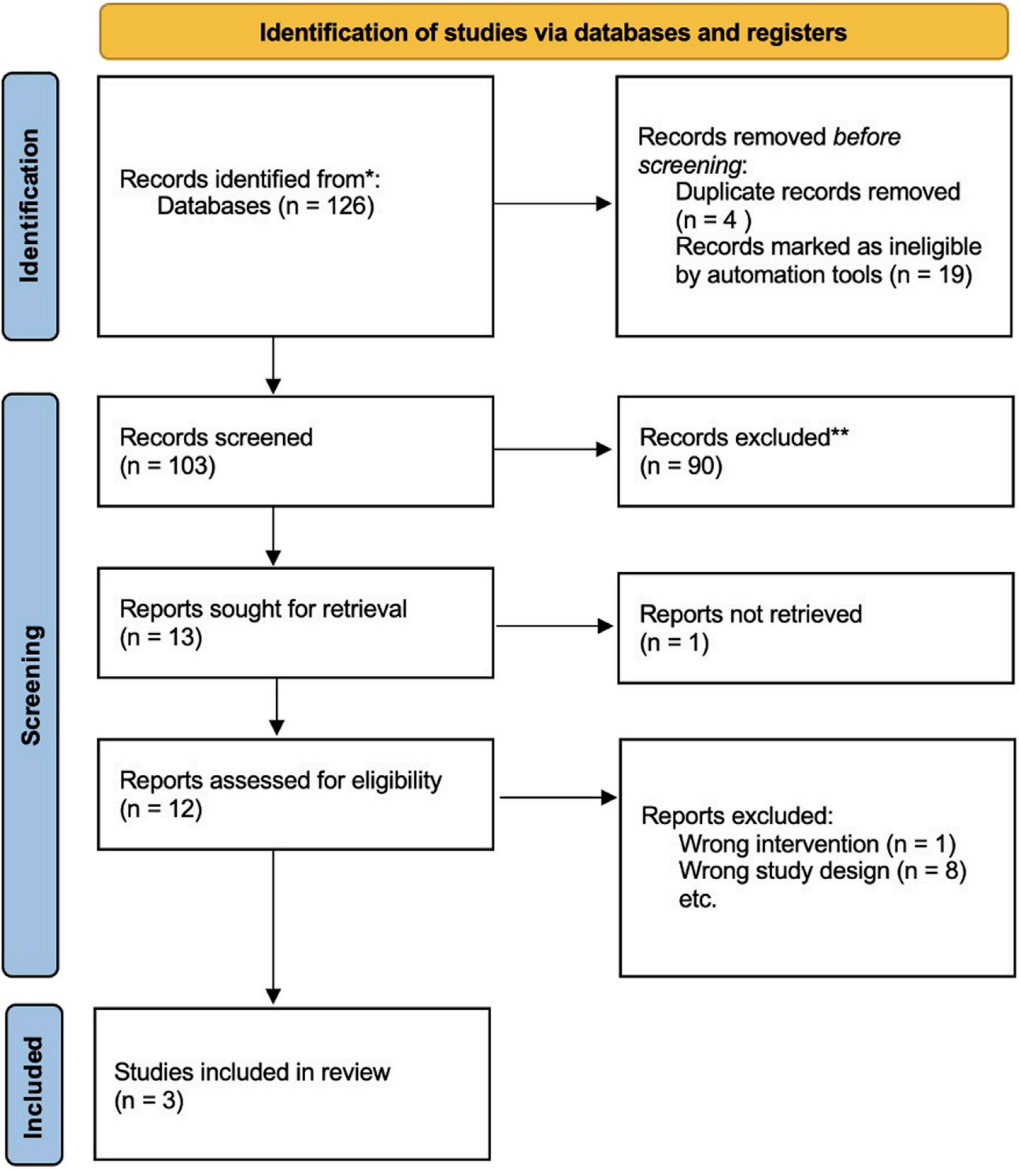
PRISMA flowchart.

Figure 1 shows the 2020 PRISMA flowchart, outlining the results of the search strategy. General characteristics of the studies and the groups studied are listed in Table 1. Overall MINORS score are shown in Table 1. The results of the quality assessment of all included studies based on the GRADE approach are presented in the Supplementary Material (Supplementary Table S2).

**TABLE 1:** Selected studies and patients characteristics reporting the robotic eTEP vs TARM approaches.

Author	Country	Year of Publication	Enrollment (Years)	Type of Study	Total Patients	Patients in eTEP Group	Patients in TARM Group	Approach	MINORS overall score
[26]	USA	2024	2015–2021	Observational (Retrospective)	96	60	36	Both lateral	23
[27]	Belgium	2022	2019–2022	Observational (Retrospective)	48	34	14	eTEP: caudal; TARM: lateral	19
[28]	USA	2020	2013–2019	Observational (Propensity Score Matched)	164	82	82	Both lateral	22

Author	Age eTEP (Mean ± SD)	Age TARM (Mean ± SD)	Sex Ratio (M/F) % eTEP	Sex Ratio (M/F) % TARM	BMI (kg/m^2^) eTEP (Mean ± SD)	BMI (kg/m^2^) TARM (Mean ± SD)	Primary/Incisional Hernia, n (%) eTEP	Primary/Incisional Hernia, n (%) TARM
[26]	57.35 ± 11.2	57.47 ± 12.0	28 (46.6%)/32 (53.3%)	18 (50%)/18 (50%)	30.47 ± 4.7	33.24 ± 6.5	28 (46.6)/32 (53.3)	16 (44.4)/20 (55.5)
[27]	62 (20–90)	54 (37–81)	19 (56)/15 (44)	12 (86)/2 (14)	31 (20–42)	30 (26–38)	20 (59)/14 (41)	9 (64)/5 (36)
[28]	57 ± 14.5	56.6 ± 13.2	42 (51.2)/40 (48.8)	39 (47.6)/43 (52.4)	31.8 ± 7.2	32.5 ± 6.6	32 (39)/50 (61)	28 (34.1)/54 (65.9)

eTEP, extended (or enhanced) view totally extraperitoneal; TARM, transabdominal retromuscular; MINORS, Methodological Index for Non-randomised Studies; SD, standard deviation; M, male; F, female; BMI, body mass index.

Of the three studies [26–28] included in the review, all were retrospective. Of the 308 patients, 176 were in the robotic eTEP group, while 132 were in the robotic TARM/TARUP group. Study characteristics and patient variables are listed in Table 1. Additional details on preoperative and intraoperative patient characteristics are available in Supplementary Tables S3, S4.

### Meta-Analysis

#### Primary Outcomes

**FIGURE 2:**
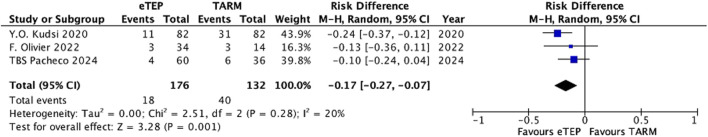
Total complications.

All studies analyzed the total postoperative complications [26–28]. Data are listed in Table 2. The risk difference between the two groups was statistically significant in favor of r-eTEP (RD: -0.17; 95% CI: -0.27 to −0.07; p = 0.001; I^2^ = 20%) (Figure 2).

**TABLE 2:** Postoperative characteristics and short-term postoperative outcomes.

Author	LOS (days) eTEP, mean (SD)	LOS (days) TARM, mean (SD)	IO complications n (%) eTEP	IO complications n (%) TARM	Drain placement eTEP, n (%)	Drain placement TARM, n (%)	SSI eTEP, n (%)	SSI TARM, n (%)	Seroma eTEP, n (%)	Seroma TARM, n (%)	Hematoma eTEP, n (%)	Hematoma TARM, n (%)
[26]	0 (0–1)*	1 (1–1)*	NR	NR	NR	NR	0	1 (2.7)	5 (8)	4 (11)	NR	NR
[27]	1 (1–3)*	1 (1–5)*	0 (0)	0 (0)	0 (0)	0 (0)	0	0	0	2 (14)	0	0
[28]	0 (0–5)*	0 (0–20)*	0 (0)	4 (4.9)	2 (2.4)	1 (1.2)	1 (1.2)	4 (4.9)	3 (3.7)	11 (13.4)	1 (1.2)	0 (0)

Readmission (30d) eTEP	Readmission (30d) TARM	Tot Compl eTEP	Tot Compl TARM	Minor complication (CD I/II) eTEP	Minor complication (CD I/II) TARM	Major complication (CD III/IV) eTEP	Major complication (CD III/IV) TARM	FU (months) median (range) eTEP	FU (months) median (range) TARM	Recurrence eTEP, n (%)	Recurrence TARM, n (%)
0 (0)	1 (2.7)	24 (40)	14 (38.9)	NR	NR	NR	NR	NR	NR	0	0
0 (0)	0 (0)	3 (8.8)	3 (21.4)	2 (6)	3 (21)	1 (3)	0 (0)	15 (3–29)	35 (29–37)	0	0
4 (4.9)	4 (4.9)	11 (13.4)	31 (37.8)	9 (11)	25 (30.5)	2 (2.4)	6 (7.3)	NR	NR	0 (0)	0 (0)

LOS, length of stay; *median (range); IO, intraoperative; SSI, surgical site infection; Tot. Compl., total complications; CD, Clavien-Dindo; FU, follow-up; eTEP, extended (or enhanced) view totally extraperitoneal; TARM, transabdominal retromuscular.

**FIGURE 3:**
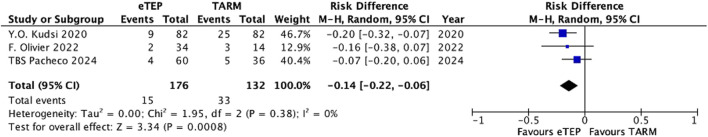
Minor complications.

All studies examined minor postoperative complications [26–28]. The risk difference between the two groups was statistically significant in favor of r-eTEP (RD: -0.14; 95% CI: -0.22 to −0.06; p = 0.0008; I^2^ = 0%) (Figure 3).

**FIGURE 4:**
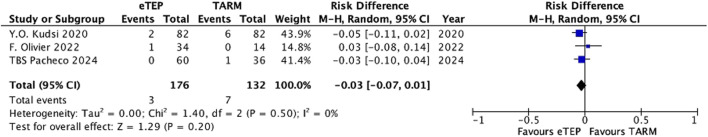
Major complications.

All studies evaluated major postoperative complications [26–28]. The difference in risk between the two groups was not statistically significant (RD: -0.03; 95% CI: −0.07 to 0.01; p = 0.20; I^2^ = 0%) (Figure 4).

**FIGURE 5:**
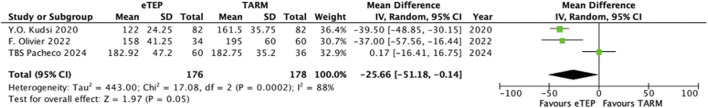
Operative time.

All studies analyzed the operative time [26–28]. This was 154.31 min [±38.80 min] in the r-eTEP group and 179.75 min [±45.16 min] in the r-TARM/TARUP group. The mean difference between the two groups was statistically significant in favor of r-eTEP (MD: -25.66; 95% CI: −51.18 to −0.14; p = 0.05; I^2^ = 88%) (Figure 5).

#### Secondary Outcomes

**FIGURE 6:**
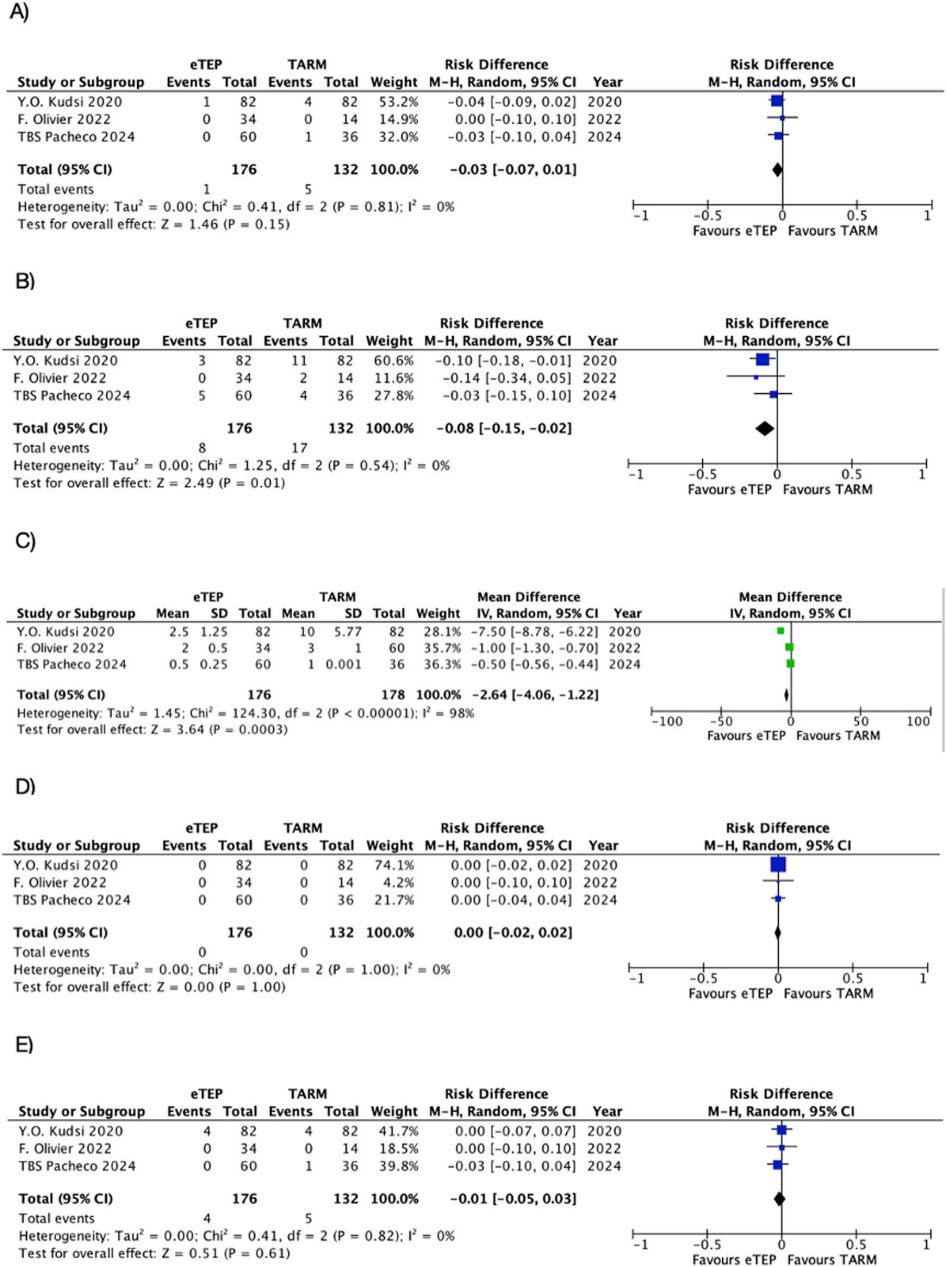
Secondary outcomes. **(A)** SSI. **(B)** Seroma. **(C)** LOS. **(D)** Recurrence. **(E)** Readmission.

All studies assessed SSI [26–28]. The difference in risk between the two groups was not statistically significant (RD: −0.03; 95% CI: −0.07 to 0.01; p = 0.15; I^2^ = 0%) (Figure 6A).

All studies analyzed seromas [26–28]. The difference in risk between the two groups was statistically significant in favor of r-eTEP (RD: −0.08; 95% CI: −0.15 to −0.02; p = 0.01; I^2^ = 0%) (Figure 6B).

All studies examined the LOS [26–28]. The mean difference between the two groups was statistically significant in favor of r-eTEP (MD: -2.64; 95% CI: -4.06 to −1.22; p = 0.004; I^2^ = 98%) (Figure 6C).

All studies analyzed short-term recurrences [26–28]. The difference in risk between the two groups was not statistically significant (RD: 0.00; 95% CI: −0.02 to 0.02; p = 1.00; I^2^ = 0%) (Figure 6D).

All studies analyzed the 30-day readmission [26–28]. The difference in risk between the two groups was not statistically significant (RD: -0.01; 95% CI: -0.05 to 0.03; p = 0.61; I^2^ = 0%) (Figure 6E).

Two studies evaluated postoperative pain using the VAS scale [26, 28]. The mean difference between the two groups was not statistically significant (MD: −0.49; 95% CI: −1.47 to 0.49; p = 0.33; I^2^ = 96%) (Supplementary Figure S2). Due to the limited number of studies and the high heterogeneity observed, we therefore decided not to include this analysis in the main manuscript, as the results are not very reliable.

#### Methodological Quality of Studies

The studies [26–28] achieved a median MINORS score of 22 (range 20.5–22.5). This result indicates a low risk of bias in the included studies. The complete MINORS scores evaluation for each study are shown in Supplementary Table S1.

## Discussion

Our meta-analysis revealed statistically significant differences in favor of r-eTEP with respect to several outcomes, including overall complications, minor complications, operative time, seroma rate, and length of stay (LOS). To date, only one other systematic review and meta-analysis has been published on this topic by Tryliskyy et al. [29] Their study included data from Olivier et al. [27] and Kudsi et al. [28], but not the recently published study by Pacheco et al. [26] In contrast to our results, their meta-analysis found no significant differences between robotic eTEP and TARM/TARUP. However, a potential limitation of their analysis was the inclusion of the study by Zaman et al. [30], comparing robotic eTEP with robotic transabdominal hernia repair with preperitoneal mesh (rTAPP, called TASM in their study) and not with a purely retromuscular approach. This methodological discrepancy may have influenced their conclusions and limited the generalizability of their results to the r-eTEP vs. r-TARM/TARUP debate.

Regarding the surgical approach for the two techniques, Kudsi et al. [28] and Pacheco et al. [26] used a lateral approach for both procedures. In contrast, Olivier et al. [27] employed a lateral approach for rTARM/TARUP and a caudal approach for r-eTEP, referred to as inverted TEP (iTEP) in their study. The authors state that the iTEP technique represents a robotic Rives-Stoppa repair, in which access to the retrorectus space is achieved through the suprapubic preperitoneal region, utilizing a single-docking approach.

Our analysis showed a statistically significant reduction in overall and minor complications in favor of r-eTEP, while the incidence of major complications remained comparable between groups. Kudsi et al. [28] reported a complication rate of 13.4% in the r-eTEP group compared to 37.8% in the r-TARM/TARUP group, with seromas accounting for 44% of minor complications in the latter cohort. Several factors may explain this discrepancy. One possible explanation is the learning curve effect, as r-TARM/TARUP was performed earlier in the surgeons’ experience, which may have contributed to the higher rate of minor complications. In addition, differences in mesh material may have played a role, as polyester mesh was used in 43.9% of r-TARM/TARUP cases, while polypropylene mesh was used in 61% of r-eTEP cases, which may have influenced complication rates. Another factor could be the extent of adhesiolysis required in the r-TARM/TARUP group. In 45.1% of patients, adhesiolysis lasted more than 30 min, a known risk factor for postoperative complications [28]. These results suggest that the perceived superiority of r-eTEP over r-TARM/TARUP in terms of complications may be study-specific rather than an inherent advantage of the technique. Given these observations, further prospective, standardized studies are needed to gain a clearer understanding of the comparative safety profiles of these procedures.

Our analysis showed a statistically significant reduction in operating time in favor of r-eTEP, a result that is consistent with the studies by Kudsi et al. [28] and Olivier et al. [27] Notably, the iTEP technique used in the latter study involves a suprapubic docking approach, which may differ in efficiency and setup time compared to standard lateral docking. This variation in surgical access could have contributed to the observed differences in operative time and should be considered when interpreting pooled results.

In contrast to r-eTEP, r-TARM/TARUP requires the posterior rectus sheath to be opened and closed, which requires additional steps that prolong operation time. In addition, r-TARM/TARUP was often performed earlier in the surgeons’ learning curve, which may have impacted efficiency and technical skills. Previous studies on robotic retromuscular repair support this learning curve effect. Muysoms et al. analyzed robotic transabdominal retromuscular umbilical prosthetic repair (r-TARUP) and reported that the average time for peritoneal closure was initially 21 min, but decreased to 18 min with increased experience [13]. In addition, adhesiolysis, which was more common in the r-TARM/TARUP group, was associated with a median operative time of 20 min, which also contributed to the increased duration observed in this cohort.

Both r-eTEP and r-TARM/TARUP appear to be safe techniques in terms of the occurrence of surgical site infections (SSI), with an overall low incidence of this complication. The highest number of SSIs was reported by Kudsi et al. [28], with one event in the r-eTEP group (1.2%) and four events in the r-TARM/TARUP group (4.9%). This result could be related to the seroma rate and the extent of adhesiolysis, as both factors are known to contribute to an increased risk of infection. Extensive adhesiolysis was identified as an independent predictor of both seroma formation and SSI [31], emphasising the need for careful patient selection and surgical planning when performing robotic retromuscular repairs.

Seroma rates were comparable between the two groups. Kudsi et al. [28] reported a seroma rate of 13.4% in the r-TARM/TARUP group, a finding that may be due to the higher incidence of extensive adhesiolysis in this cohort. Notably, 45.1% of r-TARM/TARUP patients underwent adhesiolysis lasting longer than 30 min, which likely contributed to the increased seroma rate observed [31].

Our meta-analysis showed a statistically significant difference in LOS between the two techniques. In fact, Pacheco et al. reported a shorter hospital stay in patients who underwent r-eTEP, which the authors attributed to less postoperative pain in this group. This finding suggests that differences in pain perception and recovery protocols may influence the length of hospital stay. One possible explanation for this difference is the use of additional ports in r-TARM/TARUP and injury to the parietal peritoneum, which is highly innervated and particularly sensitive to pain [32]. Only two studies in our analysis reported postoperative pain data using the VAS scale, and although our meta-analysis showed no statistically significant difference, the large heterogeneity and limited number of studies make definitive conclusions difficult. Given the conflicting views in the literature [28, 33], further research is needed to clarify the relationship between surgical procedure, postoperative pain and LOS.

Regarding the recurrence, all studies reported the occurrence of events and no statistically significant difference was found. However, only the study by Olivier et al. [27] provided data on long term follow-up, with an approximate duration of 3 years and no recurrences. The study by Kudsi et al. [28] reports a 90-day follow-up, while Pacheco et al. [26] provide 90-day follow-up data for 40% of the study population. Therefore, only short-term considerations regarding recurrence could be deemed reliable. A factor that may influence recurrence rates is the mesh coverage area, as it can affect tissue engagement and mechanical stability, potentially reducing the risk of recurrence. Our results reveal a notable difference between the two studies reporting this data. Kudsi et al. found that both techniques allowed for mesh placement with similar areas (median size of approximately 300 cm^2^), whereas Pacheco et al. reported that r-eTEP enabled the placement of significantly larger meshes (mean size of approximately 700 cm^2^). The authors of this latter study attribute this disparity to the wider dissection area achieved with r-eTEP, which extends from the subxiphoid region to the Retzius space and, bilaterally to the EIT Ambivium [34]. In contrast, they state that the dissection area in r-TARM/TARUP is more restricted due to the ipsilateral incision, limiting lateral dissection. Several other studies in the literature reporting experiences with robotic eTEP show mesh areas much closer to those reported by Kudsi et al. [14, 17, 35] In principle, despite differences in access methodology, both techniques should allow for the same extent of dissection within the retrorectus space. A significant discrepancy in mesh size and, consequently, in the dissection area may instead be attributed to the fact that r-TARM/TARUP was performed earlier in the surgeon’s learning curve, potentially introducing bias into this data [26]. Further studies with longer follow-up periods are needed to confirm whether these differences translate into significant long-term benefits.

This meta-analysis is limited by the number of studies included and their retrospective design, introducing selection bias and variability in surgical technique, patient selection, and perioperative management. The lack of randomized trials limits the ability to draw definitive conclusions. It should be noted that the anatomical location of seromas (retromuscular vs. subcutaneous) was not reported in the included studies. This lack of granularity limits the ability to fully interpret the clinical significance of this complication, particularly in the r-TARM/TARUP group where higher rates were observed. Significant heterogeneity was observed in operative time and LOS, likely influenced by surgeon experience, institutional protocols, and the learning curve effect, as r-TARM/TARUP was often performed earlier. Differences in mesh coverage area across studies raise concerns about standardization in technique.

Given the existing uncertainty and clinical balance, there is currently insufficient evidence to definitively recommend one technique over the other for robotic abdominal wall surgery. Further high-quality, prospective studies focusing on patient-reported outcomes and long-term recurrence rates are needed to inform surgical decisions and optimize patient care.
