# Correction: Comparison of robot-assisted enhanced-view totally extraperitoneal (eTEP) and transabdominal retromuscular (TARM aka TARUP) ventral hernia mesh repair: a systematic review and meta-analysis

**DOI:** 10.3389/jaws.2026.16656

**Published:** 2026-08-03

**Authors:** Francesco Brucchi, Annabelle De Troyer, Richard Sassun, Gianlorenzo Dionigi, Filip Muysoms

**Affiliations:** 1 General Surgery Residency Program, University of Milan, Milan, Italy; 2 Division of Surgery, Istituto Auxologico Italiano, Istituto di Ricovero e Cura a Carattere Scientifico (IRCCS), Milan, Italy; 3 Department of General Surgery, Ziekenhuis Aan de Stroom (ZAS) Antwerp, Antwerp, Belgium; 4 Colorectal Surgery Unit, Fondazione IRCCS Istituto Nazionale Dei Tumori, Milan, Italy; 5 Department of Pathophysiology and Transplantation, University of Milan, Milan, Italy; 6 Department of Surgery, Maria Middelares Ghent, Ghent, Belgium

**Keywords:** robot-assisted, robotic, eTEP, TARM, TARUP

Masurkar (2020) (reference 21) was not cited in the article. The citation has now been inserted in the section **Introduction**, 4^th^ paragraph and should read:

“Both the robotic eTEP and transabdominal retromuscular (TARM) **[21]** techniques allow access to the retrorectus space, enabling a minimally invasive alternative of the Rives-Stoppa repair. However, each method offers unique advantages and potential risks, emphasizing the need for direct comparison of their outcomes”.

The original version of this article has been updated.

