# Supplementary material for: Comparison of Robot-assisted Enhanced-view Totally Extraperitoneal (eTEP) and Transabdominal Retromuscular (TARM aka TARUP) Ventral Hernia Mesh Repair: A Systematic Review and Meta-Analysis
Source: J Abdom Wall Surg. 2025 Jul 4;4:14723. doi: 10.3389/jaws.2025.14723 (PMC12270933; doi:10.3389/jaws.2025.14723)
Supplement: Supplementary file 1 [file Supplementaryfile1.docx]

**Fig. 1s: Detailed search strategy**

**Pubmed (72 results)**

("Hernia, Ventral"[MeSH] OR "Incisional Hernia"[MeSH] OR ventral hernia*[tiab] OR incisional hernia*[tiab])

AND

("Surgical Mesh"[MeSH] OR "Herniorrhaphy"[MeSH] OR mesh repair*[tiab] OR prosthetic mesh*[tiab] OR mesh implantation*[tiab] OR hernia repair*[tiab])

AND

("Minimally Invasive Surgical Procedures"[MeSH] OR "Laparoscopy"[MeSH] OR "Robotic Surgical Procedures"[MeSH] OR minimally invasive[tiab] OR laparoscopic[tiab] OR robotic surgery[tiab])

AND

("Extended Totally Extraperitoneal"[tiab] OR eTEP[tiab] OR "Transabdominal Retromuscular"[tiab] OR TARM[tiab] OR TARUP[tiab] OR extraperitoneal hernia repair[tiab])

AND

("Randomized Controlled Trial"[Publication Type] OR "Controlled Clinical Trial"[Publication Type] OR "Cohort Studies"[MeSH] OR "Case-Control Studies"[MeSH] OR "Prospective Studies"[MeSH] OR "Retrospective Studies"[MeSH] OR randomized controlled trial*[tiab] OR RCT*[tiab] OR controlled trial*[tiab] OR prospective cohort*[tiab] OR retrospective cohort*[tiab] OR case-control[tiab] OR observational study[tiab])

**Cochrane library (38 results)**

(MeSH descriptor: [Hernia, Ventral] OR MeSH descriptor: [Incisional Hernia] OR ventral hernia OR incisional hernia)

AND

(MeSH descriptor: [Surgical Mesh] OR MeSH descriptor: [Herniorrhaphy] OR mesh repair OR prosthetic mesh OR hernia repair)

AND

(MeSH descriptor: [Minimally Invasive Surgical Procedures] OR MeSH descriptor: [Laparoscopy] OR MeSH descriptor: [Robotic Surgical Procedures] OR laparoscopic OR robotic surgery)

AND

(extended totally extraperitoneal OR eTEP OR transabdominal retromuscular OR TARM OR transabdominal retromuscular preperitoneal OR TARUP)

AND

(MeSH descriptor: [Randomized Controlled Trial] OR MeSH descriptor: [Controlled Clinical Trial] OR MeSH descriptor: [Cohort Studies] OR MeSH descriptor: [Case-Control Studies] OR randomized controlled trial OR RCT OR prospective cohort OR retrospective cohort OR observational study)

**Embase (16 results)**

'ventral hernia'/exp OR 'incisional hernia'/exp OR (ventral NEXT/3 hernia) OR (incisional NEXT/3 hernia)

AND

'surgical mesh'/exp OR 'herniorrhaphy'/exp OR (mesh NEXT/3 repair) OR (prosthetic NEXT/3 mesh)

AND

'minimally invasive surgery'/exp OR 'laparoscopy'/exp OR 'robotic surgery'/exp OR (minimally NEXT/3 invasive) OR laparoscopic OR 'robotic surgery'

AND

('extended totally extraperitoneal' OR eTEP OR 'transabdominal retromuscular' OR TARM OR 'transabdominal retromuscular preperitoneal' OR TARUP)

AND

'randomized controlled trial'/exp OR 'controlled clinical trial'/exp OR 'cohort study'/exp OR 'case control study'/exp OR 'prospective study'/exp OR 'retrospective study'/exp OR (randomized NEXT/3 controlled NEXT/3 trial) OR RCT OR (prospective NEXT/3 cohort) OR (retrospective NEXT/3 cohort) OR 'observational study'

**Table 1s: MINORS score**

|  | **Kudsi 2020** | **Olivier 2022** | **Pacheco 2024** |
| --- | --- | --- | --- |
| **A clearly stated aim** | **2.0** | **2.0** | **2.0** |
| **Inclusion of consecutive patients** | **2.0** | **1.0** | **2.0** |
| **Prospective data collection** | **2.0** | **2.0** | **2.0** |
| **Endpoints appropriate to the aim of the study** | **2.0** | **2.0** | **2.0** |
| **Unbiased assessment of the study endpoint** | **1.0** | **1.0** | **2.0** |
| **Follow-up period appropriate to the study aim** | **2.0** | **2.0** | **2.0** |
| **Loss to follow-up less than 5%** | **2.0** | **2.0** | **2.0** |
| **Prospective calculation of study size** | **1.0** | **0.0** | **1.0** |
| **Comparability of groups (for comparative studies)** | **2.0** | **2.0** | **2.0** |
| **Statistical analysis appropriate for the study design** | **2.0** | **2.0** | **2.0** |
| **Adequate control group** | **2.0** | **1.0** | **2.0** |
| **Baseline equivalence of groups** | **2.0** | **2.0** | **2.0** |
| **Total Score** | **22.0** | **19.0** | **23.0** |
| **Risk of Bias** | **Low Risk of Bias** | **Moderate Risk of Bias** | **Low Risk of Bias** |

**Table 2s: GRADE assessment**

| Outcome | Risk of Bias | Inconsistency | Indirectness | Imprecision | Publication Bias | GRADE Quality |
| --- | --- | --- | --- | --- | --- | --- |
| Total complications (favoring r-eTEP) | Serious (Retrospective, selection bias) | Serious (Findings changed after sensitivity analysis) | Not serious (Direct comparison) | Not serious (Confidence intervals narrow) | Likely (Only retrospective studies available) | Low |
| Minor complications (favoring r-eTEP) | Serious (Single-study influence) | Serious (Results varied significantly after excluding Kudsi et al.) | Not serious (Direct comparison) | Serious (Findings depend on single study exclusion) | Likely (Few studies available) | Low |
| Major complications (no difference) | Moderate (Retrospective design, but consistent findings) | Not serious (Findings were similar across studies) | Not serious (Direct comparison) | Serious (Low event rate) | Unlikely (Well-reported outcome) | Low |
| Operative time (favoring r-eTEP) | Moderate (Learning curve effects) | Not serious (Consistent findings) | Not serious (Same endpoint measured) | Not serious (Consistently reported) | Unlikely (Objective measurement) | Low |
| Seroma rate (favoring r-eTEP after sensitivity analysis) | Serious (Initial heterogeneity, results changed after sensitivity analysis) | Serious (Initial high heterogeneity, but resolved after exclusion) | Not serious (Expected complication) | Serious (Variability in definitions) | Possible (Selective reporting) | Low |
| Surgical site infections (SSI) (no difference) | Moderate (Low event rate, but consistent findings) | Not serious (Consistent findings) | Not serious (Well-defined outcome) | Serious (Low event rate, possible underreporting) | Possible (Studies may underreport) | Low |
| Length of stay (LOS) (no significant difference) | Serious (High heterogeneity, conflicting results) | Serious (Different trends reported) | Not serious (Standard metric) | Serious (Different discharge criteria) | Likely (Institutional variation) | Low |
| Recurrence (no difference) | Moderate (Limited long-term follow-up) | Not serious (Similar findings) | Not serious (Direct measurement) | Serious (Only two studies provided follow-up) | Possible (Limited follow-up, possible underreporting) | Low |

**Table 3s: Preoperative hernia characteristics**

| **Author** | **Recurrent Hernia, n(%) eTEP** | **Recurrent Hernia, n(%) TARM** | **EHS Classification eTEP, n** | **EHS Classification TARM, n** | **Defect Width (cm) eTEP (Mean ± SD)** | **Defect Width (cm) TARM (Mean ± SD)** | **Associated Diastasis, n(%) eTEP** | **Associated Diastasis, n(%) TARM** |
| --- | --- | --- | --- | --- | --- | --- | --- | --- |
| **Pacheco et al. 2024** | 13 (21.6) | 13 (36.1) | NR | NR | 4.9±2.6 | 5.10±1.4 | 52 (86.6) | 27 (75) |
| **Olivier et al. 2022** | NR | NR | M1- 1; M2- 4; M3- 33 | M1- 1; M2- 3; M3- 13 | 30 (10–50)* | 30 (20–50)* | 34 (100) | 14 (100) |
| **Kudsi et al. 2020** | 33 (40.2) | 35 (42.7) | NR | NR | 18.8 (12.5–75.4)* | 23.5 (9.4–70.6)* | NR | NR |

**Table 4s: Intraoperative hernia characteristics**

| **Author** | **Mesh Area (cm²) eTEP (Mean ± SD)** | **Mesh Area (cm²) TARM (Mean ± SD)** | **Mesh Material eTEP** | **Mesh Material TARM** | **Mesh Fixation eTEP** | **Mesh Fixation TARM** | **Skin-to-Skin Time (min) eTEP (Mean ± SD)** | **Skin-to-Skin Time (min) TARM (Mean ± SD)** | **Conversion eTEP, n(%)** | **Conversion TARM, n(%)** | **Bleeding eTEP (ml), median (range)** | **Bleeding TARM (ml), median (range)** |
| --- | --- | --- | --- | --- | --- | --- | --- | --- | --- | --- | --- | --- |
| **Pacheco et al. 2024** | 724.56±193.7 | 329.06±70.3 | Polipr. 60 (100) | Polipr. 24 (66.6)  Poliest. 12 (33.3) | Glue: 55 (91.2) | Suture: 29 (80) | 182.92±47.2 | 182.75±35.2 | 0 (0) | 0 (0) | NR | NR |
| **Olivier et al. 2022** | NR | NR | PVDF-2 (6); Poliest.-32 (94) | PVDF-12 (86); Poliest.-2 (14) | No | No | 158 (95–260)* | 195 (90–330)* | 0 (0) | 0 | 0 (0) | 0 (0) |
| **Kudsi et al. 2020** | 300 (225–600)* | 330 (225–625)* | Polipr. 50 (61); Poliest. 6 (7.3); ePTFE 26 (31.7) | Polipr. 27 (32.9); Poliest. 36 (43.9); ePTFE 19 (23.2) | 4 (4.9) | 32 (39) | 122 (88–185)* | 161.5 (97–240)* | 0 (0) | 0 (0) | 5 (5–20)* | 5 (5–15)* |

***median (range)**

**Fig. 2s: Pain meta-analysis**

**
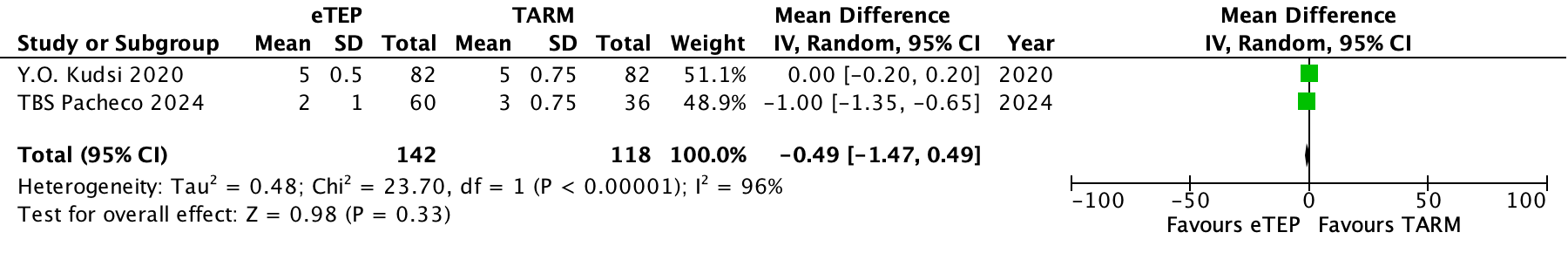
**
